# Underlying factors and challenges of implementing the urban family physician program in Iran

**DOI:** 10.1186/s12913-021-07367-3

**Published:** 2021-12-14

**Authors:** Mohammad Hossein Mehrolhassani, Vahid Kohpeima Jahromi, Reza Dehnavieh, Mahla Iranmanesh

**Affiliations:** 1 Department of Health Management, Policy and Economics, Faculty of Management and Medical Information Sciences,, Kerman University of Medical Sciences, Kerman, Iran; 2 Research Center for Social Determinants of Health, Jahrom University of Medical Sciences, Jahrom, Iran; 3 Health Foresight and Innovation Research Center, Institute for Futures Studies in Health, Kerman University of Medical Sciences, Kerman, Iran

**Keywords:** Referral system, Urban family physician, Underlying factors, Program implementation, Iran

## Abstract

**Background:**

The family physician program was launched in 2005 in rural areas of Iran and then piloted in 2012 in the cities of Fars and Mazandaran provinces due to insufficient health coverage in these cities. However, despite its pivotal role in the health system, this program has not progressed according to the policies. This study aimed to explain the underlying factors and challenges of implementing the urban family physician program in Iran.

**Methods:**

This qualitative study was conducted on 44 policy-makers and managers at national and provincial levels selected via snowball and purposive sampling with maximum variation. The data were managed in MAXQDA 2020 and analyzed by directed content analysis. A triangulation method was adopted for this purpose.

**Results:**

A total of 10 categories, 18 sub-categories, and 29 codes were formed. Most challenges related to underlying factors included precipitancy, economic sanctions, belief in traditional medicine, belief in the expertise of previous physicians, and global ranking of countries. For program implementation, most challenges included a diversity of insurance organizations, budget allocation, referral system, electronic file, educational system, and culture building.

**Conclusions:**

The major challenges pertaining to underlying factors included international pressure for reforms and precipitancy in program implementation due to management changes. The challenges associated with program implementation included budget provision and interaction with insurance organizations. Therefore, to expand this program to other provinces in Iran, the identified factors should be carefully considered so that sufficient confidence and commitment can be guaranteed for all stakeholders.

## Background

According to the recommendations of the World Health Organization (WHO), the implementation of family physician programs is an effective strategy to improve service delivery, reduce costs, and establish equity in healthcare delivery [1]. The family physician program and referral system has been implemented since 2005 in rural areas of Iran [2]. Family physician is a person with a general practitioner degree who provides services with his or her team of graduates of family health, public health, nursing, and midwifery [3]. He or she is responsible for providing comprehensive services, service continuity, health management, research and coordination with other departments, And is obliged to provide the desired services to the target group within the service package [4].

Despite many discussions about the success [5–9] and problems [10, 11] of the rural family physician program in scientific texts, this program was also piloted in the cities of Fars and Mazandaran provinces in 2012 in accordance with Articles 32 and 35 of the Fifth Developmental Plan [12, 13]. Nevertheless, this program is still limited to these two provinces and has not expanded throughout the country [14]. Planners must, therefore, decide about the expansion of this program to other provinces based on comprehensive scientific evidence. However, few studies have been conducted on the implementation of the family physician program, and studies in Iran have principally focused on rural family physicians. Due to the infancy of the program in urban areas, few studies have examined this topic.

Alidosti Alidosti et al. referred to the role of literacy as a key underlying factor and considered information sharing and education of low-literacy people as major factors in the implementation of the family physician program [15]. Chaman et al. also showed that there are serious problems in levels 1 and 2 of the referral system, and sending records from family physicians to health centers has taken place in only 12.5% of the cases. Moreover, 53% of the patients in level 2 have been referred from general practitioners’ (GP) private offices and clinics [16]. Mehrolhassani et al. in a qualitative study identified seven underlying factors that affect the family physician program, including inadequate motivational mechanisms, poor educational effectiveness, low comprehensiveness of instructions, the inadequacy of per capita and its allocation, low efficiency of health information management systems, defects in the referral chain, and insufficient culture building [17]. A review study in 2012 showed that the system of referral and feedback of specialized physicians to level 1, patient follow-up, and completion of health records does not function properly, and the desired vision of health has not yet been institutionalized in people’s minds [18]. According to Farzadfar et al. there are seven categories of problems, including financial, cultural, educational, motivational, structural, executive, and contextual problems with the urban family physician program in Iran [19]. Jahromi et al. studied patients’ perceptions of family physicians and reported that only 29% of family physicians were aware of patients’ previous illnesses and problems, and most patients were satisfied with the physicians’ behavior. However, only 40–50% of the patients stated that the family physicians asked about their preferences and interests before prescribing medication, and also gave them sufficient explanations about the disease. Only a small number of family physicians were ready to visit patients at home at their request. In general, patients living in Mazandaran led to a better experience in all aspects of this program than living in Fars province [20].

There are many international studies on family physician programs. Still, in these countries, there is no distinction between urban and rural family physician programs [21]. Sans-Corrales et al. reviewed 356 articles and found that patients’ satisfaction and recovery were related to the access to and continuity of care, consultation time, physician-patient relationship, coordination, volume and duration of consultation, and implementation of preventive measures [22]. This study systematically reviewed articles on family physicians, but none of the selected articles were conducted in developing countries. Manca et al. in Canada examined the advantages and disadvantages of family physicians and referred to the role of contextual and executive factors in the implementation of this program. According to family physicians in Canada, this program has advantages such as the provision of comprehensive and preventive care, continuous communication with patients and families, ongoing treatment and feedback, flexibility, and up-to-date knowledge and information. However, it also has disadvantages such as a high referral rate and work pressure, low income, disrespect from specialists, shortage of resources, heavy paperwork and a large volume of required forms, high patient expectations, legal problems, and physicians’ insurance [23]. This study was conducted in Alberta, Canada, and was limited to 28 family physicians who were providing services. A study by Wang et al. in China showed that government investment in primary healthcare is insufficient and unsustainable, so providing insurance repayment, financial incentives, transparency, accountability, and improving the referral system can promote the efficiency and effectiveness of the system and enhance equity in access to services [24].

The present study aimed to evaluate the implementation of the Iranian urban family physician program with a new approach to examine the underlying factors and challenges of program implementation. The results can provide suitable strategies for planning and improving the referral system.

## Method

### Study design

This qualitative study using the directed content analysis was conducted in Kerman, southeastern Iran, in 2021. Semi-structured in-depth interviews were conducted from September 2015 to March 2017, with 44 policy-makers and managers at national and provincial levels enrolled via snowball and purposive sampling with maximum variation. The participants’ demographic characteristics are shown in Table 1. The interviews continued until no new piece of information emerged from the interviews. To ensure data saturation, two additional interviews were conducted.

**Table 1:** Demographic characteristics of the participants

Level	Interviewees	Number of interviews
National	Minister of Health	3
	Deputy Minister	5
	Member of Parliament	3
Provincial	University President	2
	Vice-Chancellor	8
	University expert	5
	Head of non-academic organizations	6
	Family doctor and team member	10
	Specialist	2
Total	44	

### Data collection

The interviewees were contacted in person or via phone or email. The participants were informed about the topic of the study, its objectives, and reasons for conducting it. If they accepted to participate, the interviews were conducted at the interviewee’s office or any places agreed upon by them [25]. The interviews were conducted by VKJ with a research background in the family physician program and based on the interview guide. All the interviews were audio-recorded and immediately transcribed to guide the subsequent interviews. Interviews lasted 20–80 min with an average time of 50 min. The interviewer also took field notes to share with the research team to discuss the initial findings, complications, and any modifications needed in the interview guide. The interviews were continued until reaching data saturation. After data saturation when no additional piece of data emerged, two new interviews were also conducted. In total, 44 face-to-face semi-structured interviews were performed.

### Data analysis

Directed content analysis was adopted to analyze the data, and a triangulation method was used for this purpose [26]. The data analysis and coding were undertaken manually. The directed content analysis approach was implemented in the following steps: First, two of the authors (MHM and MI) gained familiarity with the data by listening to the recorded interviews and reading and re-reading the transcribed data. Then, they independently coded the transcripts and generated an initial list of codes. In this stage, the initial results were presented, and disagreements were discussed until a consensus was reached in the presence of a third researcher (VKJ). Later, repeated patterns were identified and assigned to codes. After the initial coding and collation, the codes were analyzed to combine different codes into an inclusive theme. In this step, categories and sub-categories were identified. Finally, the coded statements were reviewed, refined, and collated, and the results were finalized. The transcribed documents were analyzed using MAXQDA 2020 (VERBI Software, Berlin, Germany).

### Trustworthiness of results

To ensure the trustworthiness and quality of the findings, the criteria proposed by Lincon and Guba were used [27]. To ensure the credibility of the findings, a maximum variation sampling method was employed to guarantee diversity in participants to reveal multiple perspectives about the challenges of implementing the program. Credibility was met through involvement with the data which lasted for about 7 months. Furthermore, to enhance credibility, continuous engagement was ensured with the respondents whereby the researchers provided the transcribed interviews to the participants and asked them to check the correspondence between their perspectives and the results. Multiple meetings were held among three members of the research team and an iterative approach was adopted to reach a final analysis [28]. The transferability of our qualitative findings was enhanced through the purposive sampling technique with maximum variation and a detailed description of the methods and procedures utilized. The dependability of the research was assured by an inquiry audit in which the third researcher (VKJ) was engaged in frequent sessions complementary comments in the coding process and transcript analysis. To increase confirmability, we interviewed key informants from different settings which allowed us to examine the consistency of different data sources from within the same method (triangulation of sources). To enhance reflexivity, we took field notes to enrich the data. We also attempted to limit the impact of our experiences on different stages of the study.

## Results

The findings are presented on two dimensions of underlying factors and challenges of implementing the urban family physician program in Iran. The underlying factors were classified into four categories: situational, structural, cultural, and external factors, with nine sub-categories and 15 codes (Table 2). The challenges of implementing the program were classified into six categories, nine sub-categories, and 14 codes (Table 3).

**Table 2:** Underlying and contextual factors of the urban family physician program in Iran

Dimension	Category	Subcategory	Code
Underlying factors	Situational factors	Managerial factors	Precipitancy
			Change of government
		Economic conditions	Sanctions on medication
			Economic sanctions
			Inflation
	Structural factors	Political factors	Politicization
			Change of policy-makers
		Economic factors	Employment and immigration of graduates
		Social factors	Material motives
		disorganization	Lack of plan
	Cultural factors	Religion	Gender differences
		Beliefs	Belief in traditional medicine
			Belief in the expertise of previous physicians
			People’s belief in the status of family physicians
	External factors	WHO program	World ranking of countries

**Table 3:** Factors affecting the establishment and implementation of the urban family physician program in Iran

Dimension	Category	Subcategory	Code
Establishment and implementation of the urban family physician program	Financial resources	Insurance	Diversity of insurance organizations
			Interaction between the health system and insurance organizations
			Payment and service purchase system
		Budget	Budget provision
			Budget allocation
	communication	Communication between levels and organizations	Reference system
			Intra- and inter-sectoral communication
	Information technology	Software	Electronic files
		Electronic networks	Internet
	Human resources	Education and empowerment	The education system
			Motivation
		Lack of expertise	The experience of ministers
	Information sharing and culture building	Education and preparation	Culture building
			Information sharing
	Facilities and equipment	Physical space and facilities	

### Dimension 1: underlying factors

#### Category 1: situational factors

##### Subcategory 1: managerial factors

Precipitancy in decision-making means that a decision is made before the necessary tools are provided and is implemented before being practically possible. This was evident in the participants’ statements.

"Unfortunately, in Iran, the family physician has fallen victim to the precipitancy of governments. At the end of the eighth government, the family physician program began in villages without proper infrastructure. Also, at the end of the tenth government, the program was planned to expand all over the country. The result of these hasty actions is what we now see in Fars and Mazandaran provinces" (M 4).

Change of governments: The healthcare programs in Iran are affected by the change of governments and ministers. With the departure of each government, a new minister takes the office and launches a new plan regardless of previous efforts. This has been more prominent in the family physician program, especially at its outset.

“A problem in our country is that governments are formed and ended every four years. In the first year, people are novice and begin to learn about issues. They start to work in the second and third years, and in the fourth year, they leave the office. In such a system, it is not possible to implement a longitudinal and time-consuming program such as the family physician program” (M 3).

##### Subcategory 2: economic conditions

Sanction on medication: According to the respondents, the impact of political and economic sanctions on the health sector has been more prominent in the pharmaceutical sector and medical equipment.

"I think we implemented this plan when we had a drug crisis in the country. I have been the Vice-Chancellor for Food and Medicine at the university for about 10 years now, and I can safely say that things have never been worse than the past 10 years. That means that people have been faced with a new plan, and when they wanted to get a prescription, they constantly heard that the drug was unavailable. We are under sanctions. This has had a negative effect" (M 14).

Economic sanctions: Sanctions imposed on Iran’s economy by the international community have directed the financial and economic resources of social programs to areas with greater priority. The lack of funding for the program has affected its implementation.

"Of course, the government is also dealing with financial problems... The government now runs the country with taxes, and most of the oil revenue is not available" (M 5).

"The issue of payments, medicine, salaries of executive staff and assistants, and the physician per capita have significant and direct relationships with the sanctions" (M 25).

Inflation: Iran experienced the highest inflation rate in the early years of the program implementation.

"If the family physician program is implemented properly, it will be economically beneficial for people; now, they pay 10% of the visit fee, but they used to pay 30%. The medicine is also free; but if there is inflation, it affects everything, not just health" (M 23).

#### Category 2: structural factors

##### Subcategory 1: political factors

Politicization means the removal and selection of ministers and officials by political-factional motives, and ignoring the principle of selecting people with the right expertise and appropriate characteristics in all areas, even non-political areas such as social service and economy.

"Authorities should not have a political view in the social service sector so that, when the governments change, they won't replace everyone due to their political views" (M 5).

Change of policy-makers: In addition to a change of governments as a transient factor (situational factor) contributing to the family physician program, changing policy-makers and ministers and health system unsustainability (as a structural factor) have forced plans and programs to be implemented in a short time.

"Unfortunately, after the revolution, no health minister has been in the office for two consecutive terms, and this in itself is a major factor motivating ministers to look for programs with quick returns which make everyone miss the details... Experience has shown that working on a structure while reforming a sector will yield more sustainable results" (M 7).

##### Subcategory 2: economic factors

Employment and immigration of graduates: A large population of young and educated people is an economic capital; however, it is also considered a serious and potential threat in Iran. The low employment rate of medical graduates impacts the process of planning and implementing employment programs.

"If the family physician program is implemented, medical graduates will not be unemployed" (S 2).

"In principle, the identity of GPs has been questioned. So, they either immigrate or choose other jobs, and that is their fate. Unfortunately, many GPs are unemployed" (M 12).

##### Subcategory 3: social factors

Material motives: Contrary to the approach of older physicians in providing services to the people, the formation of some kind of material motive among physicians has damaged the health system programs. The interviewees believed that this has led to discrimination and affected the urban family physician program.

"It has become difficult to meet the expectations of physicians, whether GPs or specialists. Nowadays, it takes much more to fill the mouths of physicians." (M 5)

##### Subcategory 4: disorganization

Lack of a plan: The health system does not have a well-considered plan for health, treatment, and health education.

"How were the health reform program or family physician program formed? Did top-level authorities make decisions about them? It was not a matter of sitting down, developing a program, determining the status quo and the standards, or covering the gaps in these programs" (M 7)

"The lack of a master plan (for example, a 20-25-year or a long-term program) has made our education system fail to adapt to the concept of family physician. In this situation, physicians graduate without being prepared for such programs" (M 4).

#### Category 3: cultural factors

##### Subcategory 1: religion

Gender differences: According to the interviewees, religion did not considerably affect the referrals to family physicians, and the role of religion was mostly observed in the gender differences among family physicians. Some female patients who had a male physician preferred to see a female physician.

"Many of our physicians are men. Some people, whether women or their spouses, do not want the family physician to be in the midst of their family problems, so we had a series of challenges in this regard. Some solutions were also proposed. For instance, the assistant of a male physician must be a woman (a female nurse or midwife). Another challenge is that many people do not believe in midwives or nurses, but the interesting thing is that they still do not allow a male physician to visit them" (M 35).

##### Subcategory 2: beliefs

Belief in traditional medicine:

Some people believe in traditional medicine and do not visit family physicians, which posed many barriers to the program.

"We have people in our target population who visit our colleagues who are traditional medicine specialists. Well, they have to pay all the costs out of their own pocket" (M 40).

Belief in the expertise of previous physicians:

Before the implementation of the urban family physician program, people were free to visit any physician they chose. Changing physicians created some problems by limiting the population covered.

"Unfortunately, the biggest cause of dissatisfaction among clients is that some physicians may have an effective treatment method or more experience, and almost all clients only believe in these few physicians" (M 38).

People’s belief in the status of family physicians: Another cultural issue is the status of family physicians (compared to specialists) among people. Iranian people prefer to consult specialists for most diseases and, as such, it is difficult for them to accept that they must consult a GP (in the form of a family physician) for most conditions.

"When physicians do not have power and authority within a society, people do not accept or trust them" (M 5).

#### Category 4: external factors

##### Subcategory 1: world ranking of countries

The global ranking of countries: External factors can impact health programs and systems even from outside the organization or country. An external factor emphasized by the interviewees was Iran’s ranking in the region and the world in terms of health indicators.

"Iran ranks low in the region and has a long way to go to realize the goals set by national policies suggesting that it must rank first in the region in terms of health indicators. The implementation of the family physician program has been an important effort of policy-makers to fill in the gap and reform the health system" (S 10).

### Dimension 2: establishment and implementation of the urban family physician program

With regards to establishing and implementing the family physician program, six categories, nine sub-categories, and 14 codes were extracted from the participants’ statements (Table 3).

#### Category 1: financial resources

##### Subcategory 1: insurance

Diversity of insurance organizations: Diverse insurance organizations in Iran, different policies of these insurance organizations (healthcare and social security insurances), and various insurances such as the oil industry, banks, and other companies presented obstacles to the implementation of the family physician program out the outset.

"The family physician program has been implemented mainly in countries that either followed the NHS system or had integrated insurance funds. In countries that have different funds with different policies, implementing a family physician program is very concerning" (M 5).

Interaction between the health system and insurance organizations: A barrier to the implementation of the urban family physician program is related to the health system’s interaction with insurance organizations. According to the experts and provincial documents obtained from the coordination meetings of the officials in these areas, contrary to the beginning of program implementation, coordination was later made between family physicians and insurance companies at least at the provincial and lower levels, so that the physicians were accepting the insurance cover of these companies.

"In my opinion, this coordination was great both at the provincial and the national level. The problem was that enough credit was not given to the program" (M 23).

Payment and service purchase system: Payment to the providers of urban family physician program has been defined for family physicians and their teams as “per capita”, and for levels 2 and 3 as “single payment”.

"The family physician's fee is paid as per capita. The basic per capita payment for the physician and his/her team increases per person according to special cases. However, the specialist or subspecialist's fee for inpatient services is paid as per hour" (S 1).

"If a family physician, for any reason, refers one of his/her patients to a specialist, he/she will receive 90% of the private visit fee and 100% of the public visit fee from insurance companies if the patient goes to the private sector; he/she will receive the fee for 3 public visits from insurance companies if the patients go to the public sector" (S 1).

##### Subcategory 2: budget

Budget provision: According to the senior managers of the Ministry of Health and Medical Education, the financing of this program greatly fluctuated, thereby impacting the realization of its objectives. According to the reviewed documents, the budget required for the urban family physician program was foreseen and approved in the budget law, but was not paid for years due to various reasons (including a change of ministers and governments).

"Providing resources is another issue in this area. For example, the family physician program was planned to start in 2011 but there was no budget then" (M 5).

Budget allocation: The allocation and use of the budget guarantee the implementation of the urban family physician program in the two provinces. This requires the efforts of authorities of the program, which has been absent for years impacted the program.

"The 11^th^ government that came to power did not have a plan to allocate budget to the program, and this was practically postponed until the end of the year" (M 6).

#### Category 2: communications

##### Subcategory 1: communication between levels and organizations

- Referral system: In the urban family physician program, an effective referral system ensures close communication between all three levels of the health system, including family physicians, specialists, and subspecialists. It also helps people receive the best possible care in the nearest location.

"Providing level 2 and level 3 infrastructures is very important. If level 2 refers to a specialist who is sitting in his/her office, then we must admit that the infrastructure is not ready yet" (M 24).

Another issue is the effective communication between various levels of the referral system.

"We are only referring to the second and third levels, but unfortunately we don't receive valuable feedback. I don't remember the exact number, but when I've been referring patients, I referred only five or six patients who gave me valuable feedback, which had an educational advantage for me" (M 37).

According to the family physicians, the coordination between family physicians at the first level and specialists at the second level was limited to a referral sheet, and there was no other communication.

"Specialists have virtually no communication with family physicians; they only know that the referral sheet consists of two pages, with one side filled out by the family physician and the other by them in response to the family physician" (M 38).

Inter- and intra-sectoral communication: Inter- and intra-organizational coordination at different levels is of particular importance. At the urban and provincial levels, the coordination between various deputies in medical universities, and between the family physician’s team and other departments to ensure the proper implementation of the referral system greatly helps advance the process.

"The dean of the university was definitely in the meetings that they were holding, and if he wasn’t, he would have asked some else to represent him. But the reason for the lack of coordination was that the therapists thought that it is the responsibility of the health system, which was not the case. Some coordination between us was good, though" (M 22).

#### Category 3: information technology

Advanced healthcare systems in the world are equipped with powerful and advanced information systems. Information technology is useful for the family physician and is also essential for access to an advanced healthcare system.

##### Subcategory 1: software

Electronic files: Urban family physicians need information recording software. This software provides physicians with patients’ information centrally, and provides program managers with more comprehensive information such as the medication and equipment, the type of services provided to the client, and his communication with different levels.

"A big problem of the program was the software, as the Iranians’ health software was also the software of the program. We entered the patients' information, medical history, and national ID number, and we were online. The big drawback was that the software was being upgraded, which meant that we didn't have the upgraded software when starting the program. We were saying, let’s start the program. However, when we started the program, four months later, we found out that we need this and that in the system, which was not even ready for six months. After six months when the system was finally ready, we found that the system is wrong here or does not work there." (M 23)

A lack of strong information technology infrastructure was another serious obstacle to the urban family physician, which needed to be resolved at the highest levels of the healthcare system.

"Completing and strengthening the IT infrastructure in the country is, doubtless, very important for electronic files, copying, electronic referrals, etc., and with the improvement of this system, works will be much easier" (M 12).

##### Subcategory 2: electronic networks

Internet: The existence of software and electronic records requires access to the Internet in both public clinics and private offices. The participants believed that:

"At the urban level, we had no problem in terms of hardware infrastructure in the public sector as we had prepared all these facilities before the official implementation of the program. But in the private sector, this infrastructure was not ready, and even to this day, some physicians have not done their work in their office" (M 22).

#### Category 4: human resources

The required human resources for the urban family physician program include the family physician specialist and his/her team.

##### Subcategory 1: education and empowerment

A family physician is a specialty with a generally preventive and health-oriented rather than therapeutic approach.

Education system: From the outset, the medical education system in Iran has trained GPs with an emphasis on the centralized treatment approach; therefore, it cannot easily cope with the family physician’s health-oriented approach and achieve the goals of the program.

"One of our problems is that physicians are not family physicians. We trained them to be physicians who treat, and this is not their fault. So, both the science we taught them and their role model were different. We all earn money from treatment, not health and prevention. These physicians have had no proper education on health and prevention, no proper observation of models, and even no income from health and prevention, so they just sit in their office and write prescriptions" (M 1).

Motivation

"It would be much better if, from time to time, some meetings were held for family physicians to express their problems and offer solutions. Unfortunately, this does not happen at all" (M 40).

##### Subcategory 2: poor expertise

Experience of ministers: The policy-makers of the family physician program have not had enough experience and expertise to manage the healthcare system and, consequently, the urban family physician program. Since it takes at least a few months for a minister to take the office, it takes him/her a while to figure out what the program means and what the plans are. Therefore, the lack of experience of people who are appointed as ministers can negatively affect the implementation of the program.

" Except for Dr. Pezeshkian who was the Deputy Minister, among the five Ministers of Health that we've had during the implementation of the program, no one had any experience with the country’s senior management" (M 5).

#### Category 5: information sharing and culture building

The implementation of the family physician program partly relies on information sharing and culture building. In this regard, culture building for the program among people and service providers is essential.

##### Subcategory 1: education and preparation

Culture building: To implement the urban family physician program, culture building should be actively done to orient the public’s mentality towards the program. The increased dissatisfaction and complaints about the urban family physician program may partly be related to this problem.

"If this program had been well explained to people, we would have created a good culture. If we had spent a few years explaining this program to people, we might not have faced the problems we are dealing with now" [29].

Information sharing: Insufficient sharing of information before the implementation of the program has caused many problems for the program providers, e.g., the large number of people visiting family physicians at the beginning of the program, disruption of the patient referral process, and patients’ dissatisfaction.

"Except for television, and especially the provincial television, the media did not advertise the program. Newspapers were less involved with the program. Television aired a weekly interview and presented the report or provided guidance" (M 23).

#### Category 6: facilities and equipment

##### Subcategory 1: physical space and facilities

According to the program implementers, the physical space, facilities, and equipment necessary for the implementation of the program were set in place, especially in the public sector and before program implementation.

"We had no problem with the hardware … There was no problem with the location and physical space" (M 23).

"We had no problem at the urban level in terms of hardware infrastructure in the governmental sector … We easily prepared these facilities even before the official implementation of the program” (M 22).

## Discussion

The family physician policy to establish a referral system has been the second major pivotal reform in the Iranian healthcare system. According to the findings, despite the positive steps taken to strengthen the service leveling system, this program still faces challenges that can be classified into two main categories of underlying/contextual factors, and challenges of program establishment and implementation.

### Underlying factors of the family physician program

According to Grindle and Thomas, underlying factors play a limiting or facilitating role in the development, implementation, and expansion of the urban family physician program [30]. The precipitancy in the family physician program has acted as a powerful force for national actors to put the program on the agenda. The role of contextual factors has also been reported by Koduah et al. [31]. This precipitancy has caused the urban family physician program to be implemented without the provision of the infrastructure such as educating and preparing people and service providers. Still, the WHO emphasizes that people should be educated and prepared before implementing any program [32]. Similarly, the precipitancy in policy-making and planning at the beginning of the urban family physician program has been reported by other researchers in Iran [33]. Perhaps one of the reasons for this precipitancy was the government’s inability to run a long-term program that extends to the next governments, which happened a year after the start of the program (June 2013). The change of government is, therefore, an underlying factor affecting the program. Some researchers have pointed to government change as an underlying factor in the ups and downs of health policies [29, 34]. The change of three ministers in the first 4 years since the start of the urban family physician program created considerable conflicts of interest between authorities with different political views. This issue has also been mentioned by Khayatzadeh and Takian [3, 14].

The economic condition in the early years of program implementation was another influential situational factor. Due to sanctions, the priority of family physician program in the health system was undermined; still, like other studies conducted in Iran that have addressed the impact of sanctions on the pharmaceutical market [35], the impact of sanctions on the urban family physician program have been prominent in specific sectors such as medication and inflation only.

The findings related to structural factors suggest that developmental plans and programs drawn up by one government and submitted to the parliament for approval may not be accepted; even with the approval, their implementation depends on the next government’s political view. This problem has previously been reported by an Iranian study [36]. In this situation, the health system lacks comprehensive plans. Meanwhile, the family physician program is a comprehensive and long-term plan that requires educational, managerial, and technical infrastructure to achieve its ultimate goal.

The effects of cultural factors on health policies and their prominent role have been widely reported [37, 38]. As identified in cultural access, in this study, cultural factors such as gender differences and belief in traditional medicine were among these factors, which had little effect on the program. The lack of family physicians’ communication skills knowledge about patients’ illnesses and medical history has negatively shaped patients’ beliefs about family physicians. Based on a review study, the components of physician-patient communication include the establishment of interpersonal communication and information transfer [39]. According to Krish et al., a gap in physician-patient communication was reported as contributing to patient dissatisfaction [40].

Iran’s average position in the global ranking has put pressure on health authorities and policy-makers to solve the problems, and this impact on the family physician program in Iran has also been reported in other studies [41].

### Establishment and implementation of the urban family physician program

The results concerning program establishment and implementation revealed that the program was implemented without complete provision of information sharing, cultural infrastructure, and communications. The common perception was that some of these problems would be resolved after program implementation [42]. The problems persisting in the Iranian referral system have also been reported by many researchers [40, 43, 44].

Before program implementation, sufficient coordination had been made between different levels of the referral system, but the communication between these levels was not well-established during implementation. The use of low-level capabilities of the health system to achieve its goals, such as improving the quality of services, has been reported in previous studies [45].

While launching and expanding the program, the software infrastructure for creating electronic health records was not ready, and this problem persisted for the first 3 years of this study. This problem was noted by previous studies as well [46]. A national measure taken in the health system at the end of 2016, which has played a significant role in resolving this shortcoming, has been the development of an integrated health system for Iranians.

The establishment and implementation of the program have been affected by the education and training of treatment-oriented GPs by the education system who find it difficult to understand the concept of family physician principles (health-centered). The lack of material motives for physicians was also mentioned. Family physicians’ lack of involvement in decisions related to the program is caused by this lack of motives [5].

The results concerning information sharing and culture building indicated that, in the previous system, people used to refer directly to specialists for any disease which formed a wrong culture. Factors such as the lack of trust in the family physician, excessive referral to the family physician, and dissatisfaction with the referral system are the consequences of this culture. This dissatisfaction with the urban family physician program reported in previous studies [47] and herein could partly be due to this culture. Of course, awareness-raising and training programs have also been administered to providers. In the study by Siddiqui et al., the cultural and social component was the main reason for physicians’ withdrawal from the rural family physician program in Iran [48].

The findings demonstrated that the required physical space, facilities, and equipment were ready for the establishment and implementation of the program. The readiness of the PHC infrastructure in the public sector and the significant participation of the private sector in cities seem to have been the main reasons for this success.

Researchers had difficulties in getting access to policy makers and program managers. Sometimes they had to wait up to one or 2 months to do an interview or request an interview by frequent visits to their offices. This problem was partially resolved by coordination with officials and policy makers as well as corporation of president of Kerman university.

However, this issue was not considerable when there were meetings or gatherings. As in these meetings researchers could meet managers and conduct an interview or set a timer for interviews.

Some managers and policy makers who attended an interview gave politically acceptable answers, considering their role and job responsibilities. They expressed speeches from a political point of view rather than scientific view. However, an attempt was made to reduce the problem by explaining the objectives of the research to the interviewees. **Conclusion.**

Various underlying factors affected the piloting of the urban family physician and caused the program to experience many ups and downs. The program has continued to this day despite the impact of underlying factors, but it is still limited to two provinces of Fars and Mazandaran and there is no plan to expand it to other provinces. Based on the findings, numerous underlying factors and challenges have impacted the urban family physician program implementation. As for underlying factors, globalization and international pressure for reform to improve Iran’s position in the global ranking, precipitancy, a change of policy-makers and programs, and sanction-induced economic conditions were influential factors on the implementation and continuation of the program. In terms of establishing and implementing the program, budget provision, interaction with insurance organizations, referral system, communication between different levels, and electronic health records were the effective factors. To expand the program to other provinces, policy-makers and planners are recommended to focus more on the above-mentioned factors and reformulate the program accordingly.

## Data Availability

The transcribed interviews and open coding are available from the corresponding author on a reasonable request. All the interviews were conducted, transcribed, and are accessible in the Persian language.

## Abbreviations

MHM: Mohammad Hossein Mehrolhassani
VKJ: Vahid Kohpeima Jahromi
RD: Reza Dehnavieh
MI: Mahla Iranmanesh
